# Inverted Sinonasal Papilloma Involving the Middle Ear with Evidence of Squamous Cell Carcinoma

**DOI:** 10.7759/cureus.6176

**Published:** 2019-11-18

**Authors:** Fatima Alghamdi, Nora Aldohan, Sarah Al-Otaibi, Mohammad Dababo, Eyas O Othman

**Affiliations:** 1 Otorhinolaryngology, Alfaisal University, Riyadh, SAU; 2 Otolaryngology, Alfaisal University, Riyadh, SAU; 3 Otolaryngology, King Faisal Specialist Hospital, Riyadh, SAU; 4 Anatomic Pathology, King Faisal Specialist Hospital, Riyadh, SAU; 5 Otolaryngology and Head & Neck Surgery, King Faisal Specialist Hospital, Riyadh, SAU

**Keywords:** inverted papilloma, squamous cell carcinoma, nasal mass, middle ear

## Abstract

Inverted papilloma is an uncommon benign epithelial tumor that typically affects the nasal cavity and originates from the lateral sinonasal wall. Inverted papilloma occurring in the middle ear is an even more rare and aggressive condition. We describe a case of a 76-year-old man who was treated for isolated sinonasal inverted papilloma and after 20 months he was found to have a recurrence in the nasal cavity with middle ear involvement and malignant changes consistent with nonkeratinizing squamous cell carcinoma arising from inverted papilloma. Several surgical procedures were required to remove the tumor along with radiation therapy postoperatively.

## Introduction

Sinonasal schneiderian papilloma is a benign neoplastic proliferation that originates from epithelial cells. Inverted papilloma (IP) compromises about 7% of all types of primary tumors occurring in the nasal cavity. These tumors have been reported to be locally aggressive with a high tendency of malignant transformation and recurrence particularly if incompletely resected. The occurrence of IP in the middle ear or mastoid process is a rare entity [1,2]. We have reviewed the literature from 1987 to 2019 and to the best of our knowledge, there are only 13 reported cases of primary sinonasal inverted papilloma with middle ear involvement in the English literature [3-14]. The majority of these cases show evidence of malignant transformation. Three of these cases were associated with human papillomavirus (HPV). We report one additional case and briefly discuss the literature.

## Case presentation

**Figure 1:**
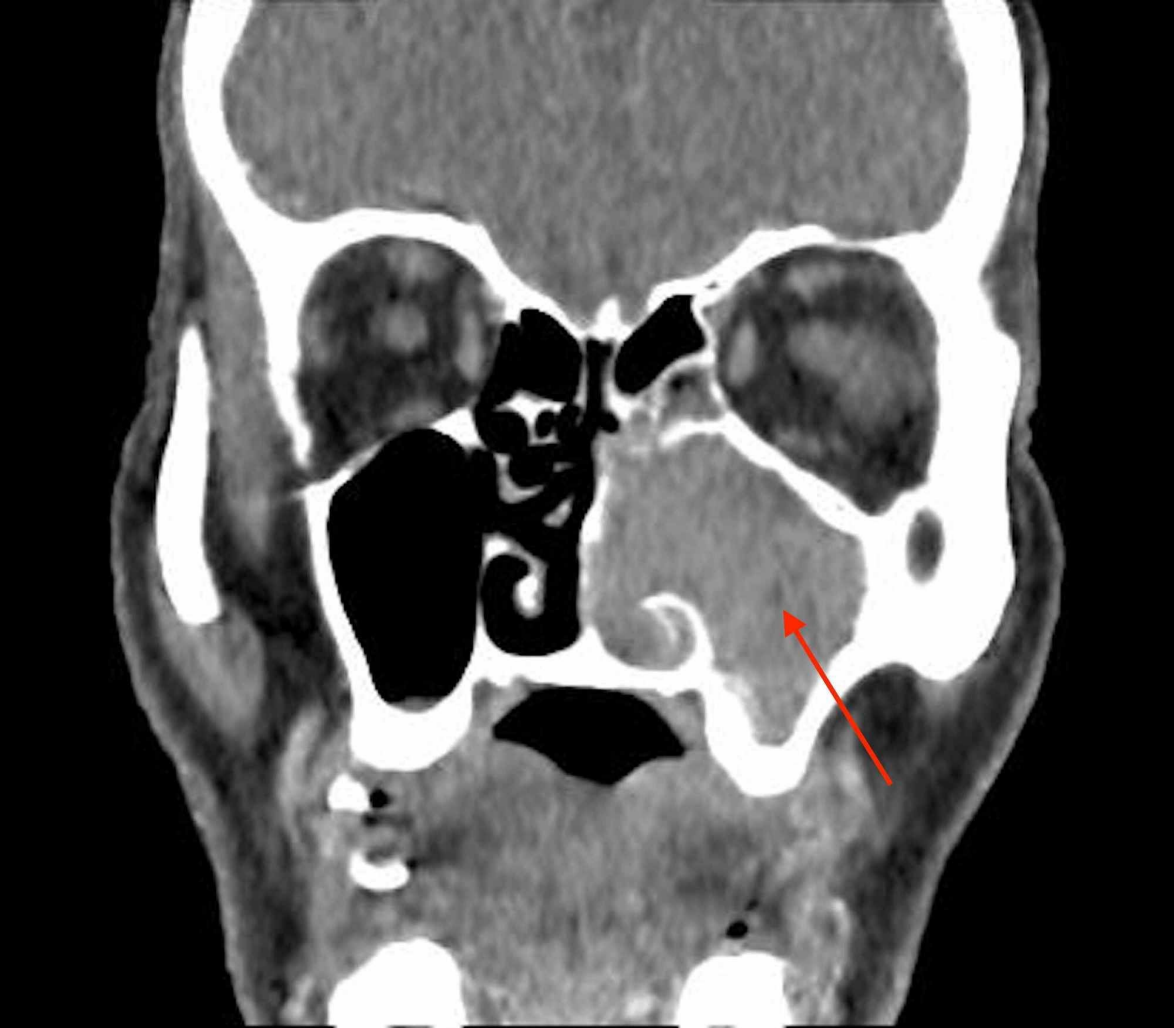
Coronal maxillofacial non-contrast computed tomography scan showing complete opacification of the left maxillary sinus & nasal cavity.

In April 2016, a 76-year-old man presented to the ear, nose & throat (ENT) clinic with a four-month history of nasal obstruction and loss of smell, on the left more than the right. There was no history of epistaxis, hearing loss, vertigo or otalgia. The ear canal, tympanic membrane and tuning fork testing was normal. On nasal endoscopy a polypoidal mass filling the left nasal cavity and extending to the right side through the nasopharynx was evident. Pathology was consistent with inverted papilloma. The remainder of the physical examination of the head and neck showed no abnormalities. Computed tomography at that time showed a locally advanced tumor of the left nasal cavity and left maxillary sinus; the temporal bone was clear (Figure 1).

**Figure 2:**
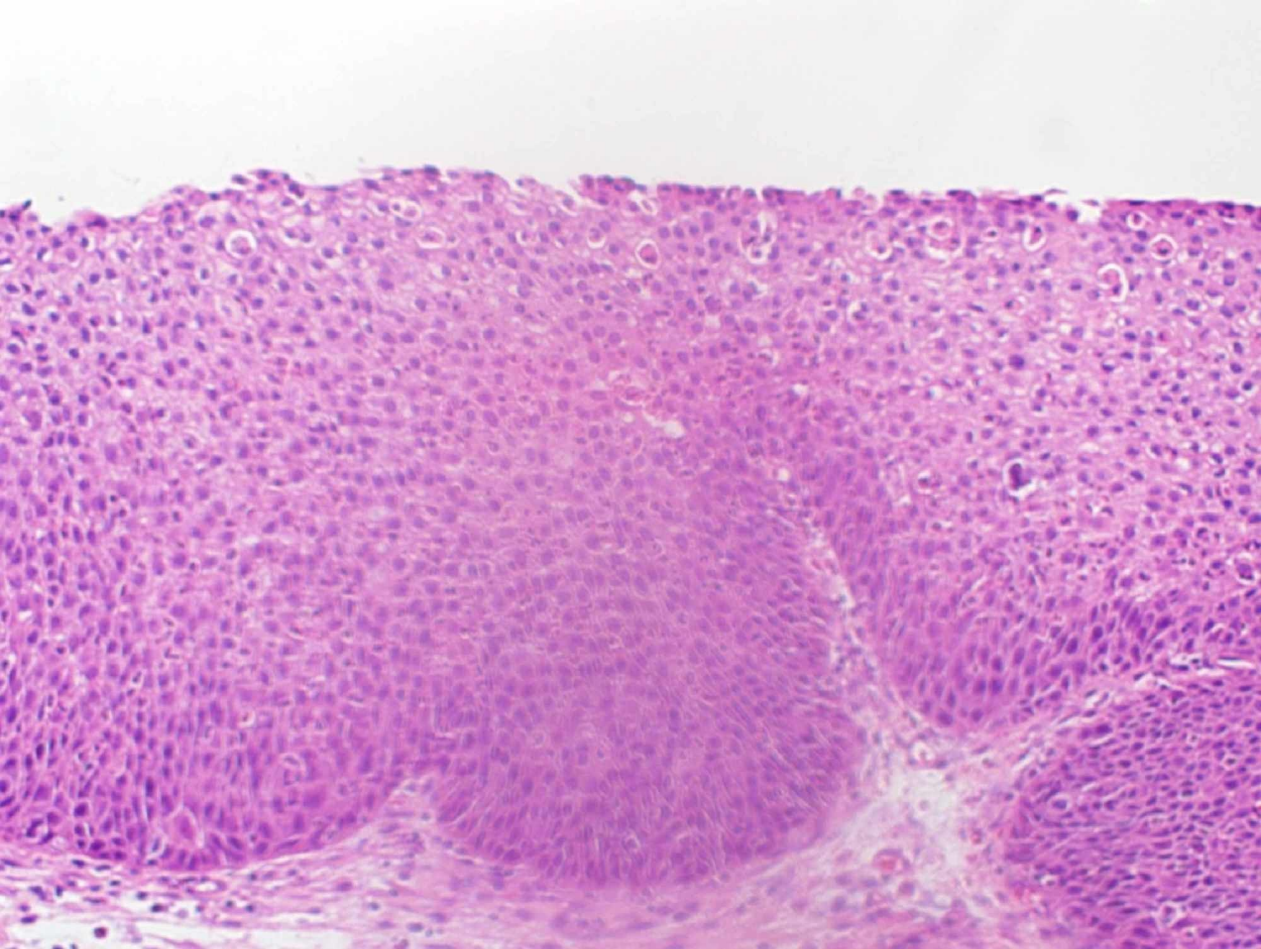
Left maxillary biopsy showing inverted papilloma with mild epithelial dysplasia.

The patient underwent a left endoscopic medial maxillectomy where the gross abnormal polypoid tissue was completely removed with histopathology findings consistent with inverted schneiderian papilloma with focal mild to moderate grade dysplasia (Figure 2).

**Figure 3:**
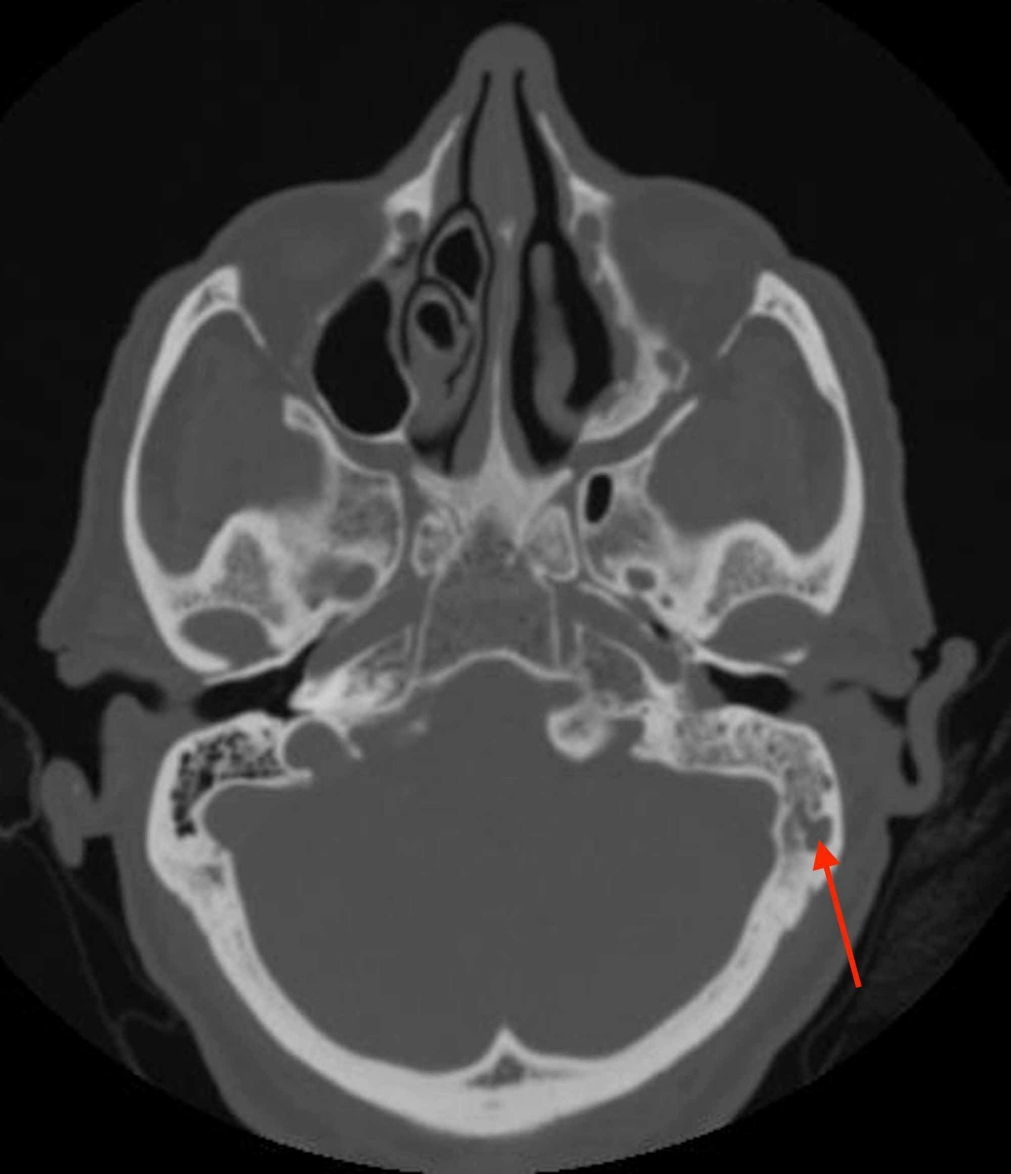
Axial temporal computed tomography. The red arrow is pointing at opacification in the left mastoid air cells.

The postoperative period was uneventful. Unfortunately, there was no follow-up until the patient presented in February 2018 after 20 months of the initial surgery with tinnitus and hearing loss in the left ear. Otoscopy showed an intact immobile left tympanic membrane. No remarkable changes in the contralateral ear. Tympanometry of the right ear revealed (type A) normal middle ear pressure and low compliance, as for the left ear it revealed flat (type B) tympanogram. Audiometry results for the right ear showed moderate sensorineural hearing loss with 48 dB pure tone average, as for the left ear results revealed severe mixed hearing loss with a pure tone average of 83 dB with an air-bone gap of 20-25 dBHL from 500 Hz to 4 KHz. Nasal endoscopy showed a small nasal mass limited to the anterior maxillary wall on the left side with an unremarkable nasopharyngeal exam. Computed tomography of the head and sinuses demonstrated complete opacification of the left middle ear cavity and mastoid air cells without evidence of erosive bony changes (Figure 3).

The patient underwent endoscopic resection of the left maxillary sinus with myringotomy and tube insertion in the left ear. A biopsy was obtained from the left middle ear after finding a fleshy mass filling the cavity. The pathology was consistent with inverted papilloma and severe epithelial dysplasia while the specimen from left maxillary sinus showed inverted papilloma with a milder form of epithelial dysplasia.

**Figure 4:**
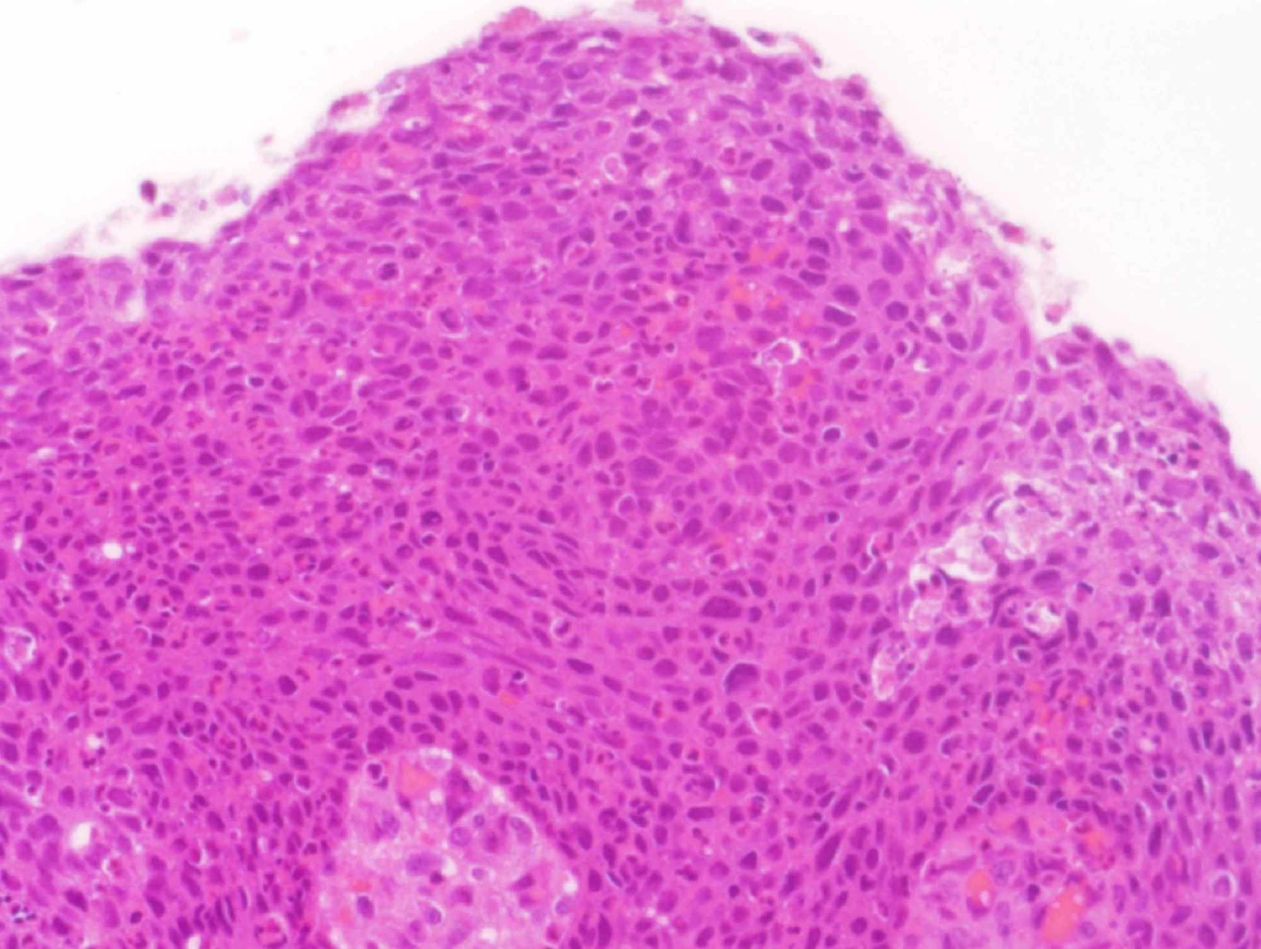
Left middle ear biopsy showing squamous cell carcinoma in situ (x20).

In May 2018, based on the above, the patient underwent a left canal wall up mastoidectomy with tympanoplasty. The tumor had filled the middle ear and extended towards the antrum and mastoid cavity. All visible tumor was completely resected, sent for pathology which showed the tissue to be consistent with nonkeratinizing squamous cell carcinoma in situ with focal micro invasion by inverted papilloma and malignant transformation (Figure 4).

**Figure 5:**
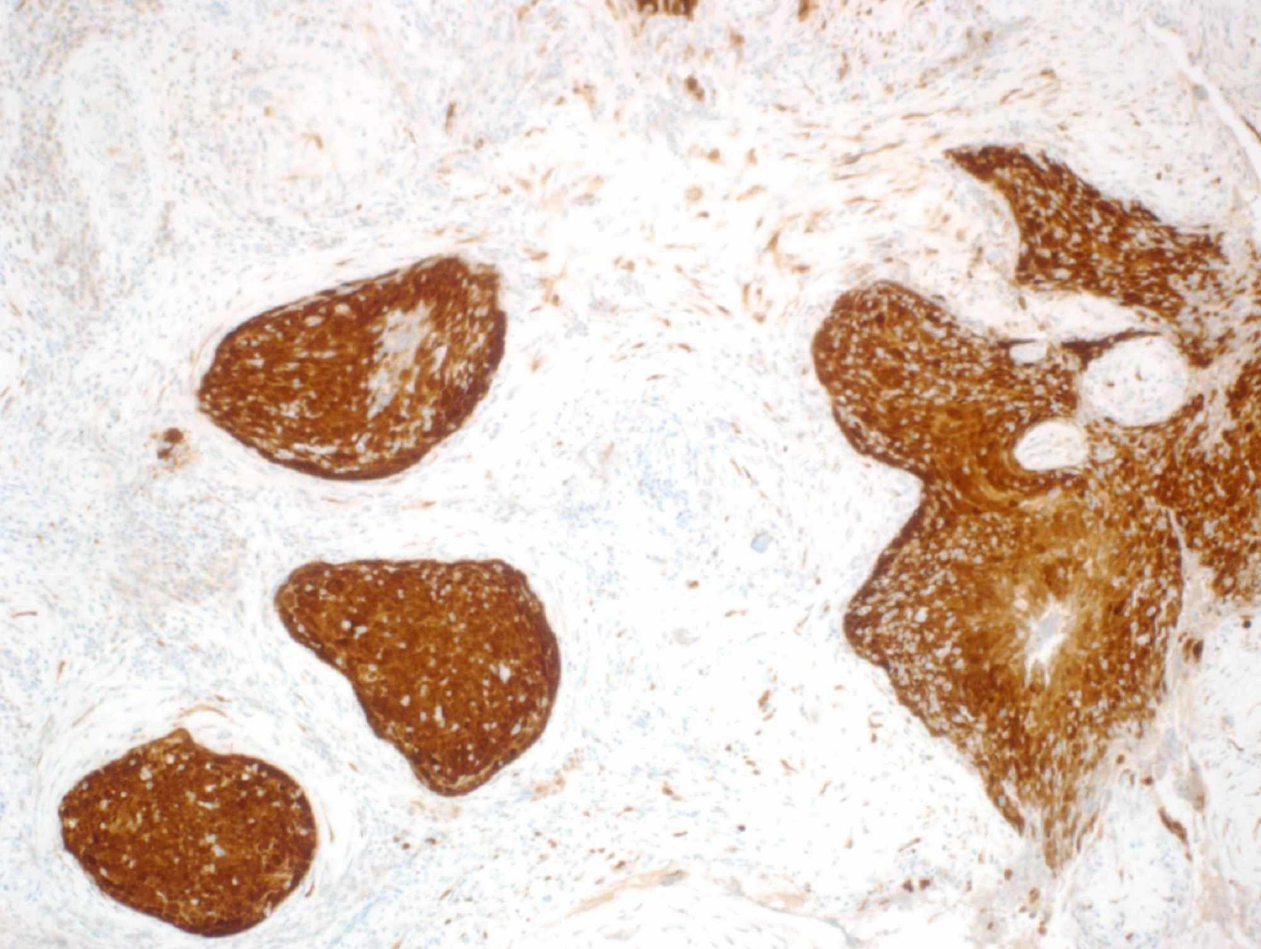
Left middle ear biopsy showing diffusely strong p16 immunohistochemistry staining.

Human papillomavirus presence was detected with immunohistochemistry and found to be strongly positive for P16 (Figure 5).

Postoperative facial nerve functional assessment was intact. The assessment was done by using the House-Brackmann grading scale which was equivalent to grade 1.

**Figure 6:**
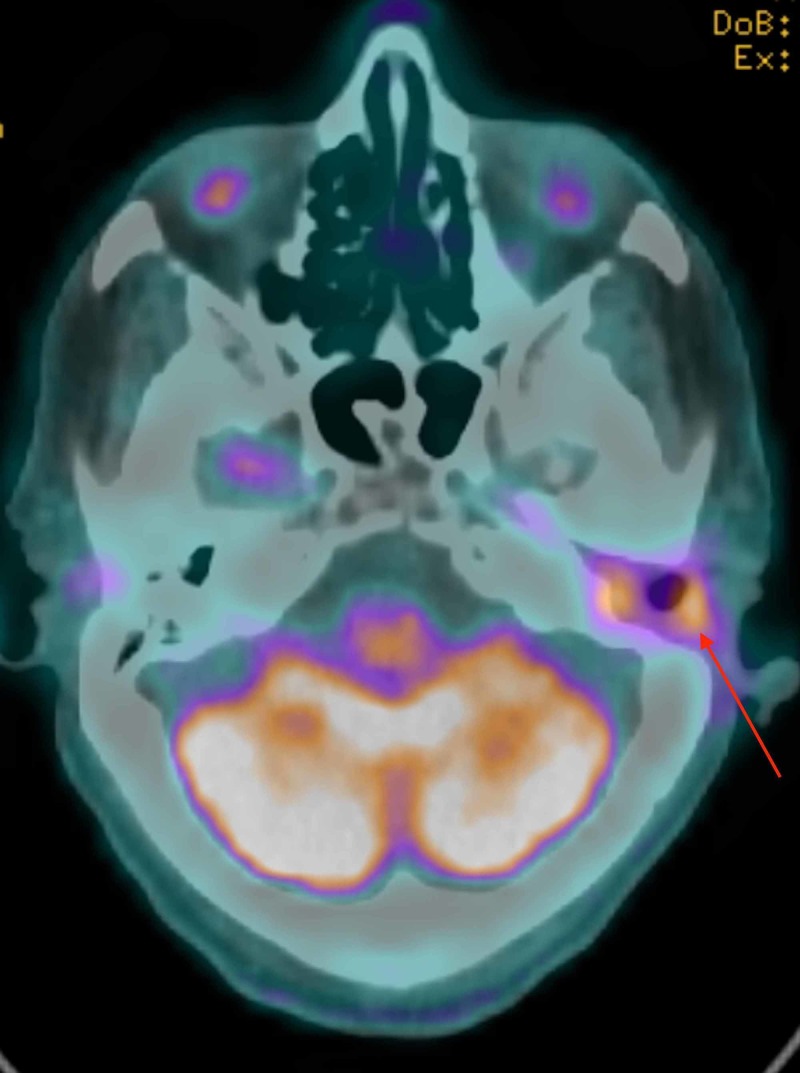
Axial head PET-CT shows FDG avid lesion located in the left external ear canal suggestive of residual active disease. FDG: Fluorodeoxyglucose

**Figure 7:**
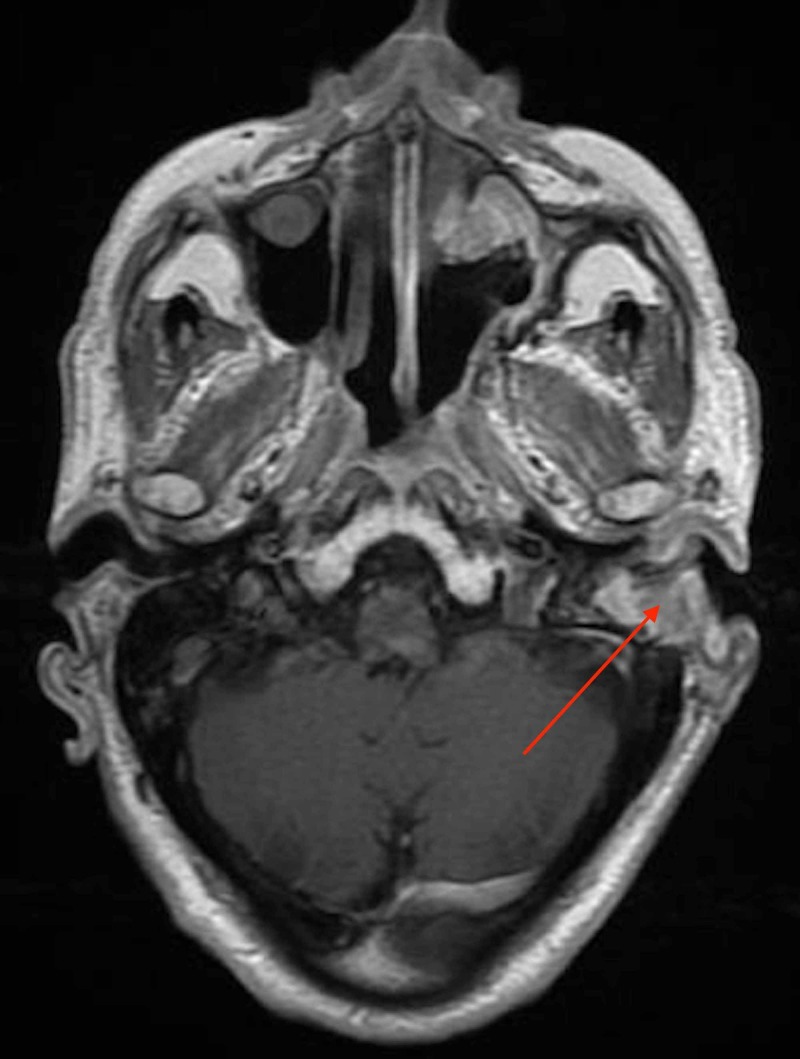
Post-operative axial MRI of the head showing interval progression of the external ear canal thickness.

In February 2019, CT scan showed erosion of the left ossicles and left tegmen tympani. The patient was admitted for a revision canal wall down mastoidectomy. The tumor had recurred and was seen in all compartments of the mastoid air cell system and the epitympanum extending to the middle ear. All tissue biopsies from the left mastoid, middle ear, and Eustachian tube showed invasive nonkeratinizing squamous cell carcinoma arising from inverted papilloma with malignant transformation. There was no involvement of nasopharynx as biopsy results were negative for neoplasm. In April 2019, the patient was referred for adjuvant radiation therapy (RT) due to residual malignant tumor in the left external auditory canal that was detected by positron emission tomography scan (PET) which showed interval progression of the fluorodeoxyglucose (FDG) avid soft-tissue attenuation that involves the left external auditory meatus (Figure 6) and magnetic resonance imagining (MRI) which showed an interval progression of left external auditory canal wall thickness (Figure 7).

The patient received a total dose of 66 Gy of radiation to the left ear and completed the course of treatment over seven weeks.

## Discussion

Schneiderian papilloma or inverted papilloma (IP) are rare benign tumors of epithelial origin. Middle ear inverted papilloma is extremely rare with 13 cases reported so far (Table 1, Appendix). Their development is theorized through one of three routes: The first and widely acceptable theory is the direct spread of primary sinonasal papilloma through the Eustachian tube; this can be justified by the unilateral pattern of involvement. The second theory is the conversion of remnant ectopic Schneiderian mucosa in the middle ear. The third theory is the embolic seeding of tumor cells outside the sinonasal tract [10]. There has been a correlation between certain HPV subtypes (6 and 11) and the development of this tumor in the sinonasal cavity. Albeit benign, this type of papilloma has a malignant potential especially if inadequately treated [15]. A meta-analysis of 31 case-controlled studies reported a statistically significant association of HPV 16 and 18 with malignant transformation of sinonasal IP [16]. Another finding amongst HPV positive cases is the high rate of malignancy reaching 75% of HPV positive cases. Several studies have linked HPV to the pathogenesis of inverted papilloma. HPV subtypes 6, 11, 16, and 18 have been isolated from the epithelium in 76% of patients with IP, possibly indicating a role of HPV vaccination [17,18]. In comparison with sinonasal IP, current data associates middle ear IP with a higher recurrence rate and malignant transformation. Reported cases that were managed by a simple tympanotomy and excision had a 100% recurrence rate in comparison to those managed by extensive mastoidectomy and/or temporal bone resection approaches which had recurrence rates as low as 39%. Recurrence after mastoidectomy or tympanomastoidectomy was noted six months to two years after surgery [19]. Adjuvant therapy in the form of radiation has been used in cases of malignant transformation, recurrence or surgically unresectable tumors. But a definitive role in the treatment of IP has yet to be established. Given the current data about the high recurrence rates and malignancy potential of Schneiderian papilloma in the middle ear, a more aggressive treatment modality with meticulous dissection and complete removal should be implemented at diagnosis.

## Conclusions

This report describes a rare case of inverted papilloma of the sinonasal cavity with middle ear involvement. Investigations revealed malignant transformation and positive HPV association. Reported cases of middle ear papilloma reveal higher recurrence rates and malignancy potential compared to those confined to the sinonasal cavity. Accordingly, an aggressive approach to the management of similar lesions is recommended along with strict close follow-up using nasal endoscopy, otoscopy and MRI to detect any recurrence.

## Appendices

**Table 1:** Reported cases of sinonasal inverted papilloma with middle ear involvement. M: Male; F: Female; FU: Follow up; SCC: Squamous cell carcinoma; RM: Radical mastoidectomy; RT: Radiotherapy; NED: No evidence of disease; AWD: Alive with disease; D: Debulking; ERM: External radical mastoidectomy; TBR: Temporal bone resection; RTM: Radical tympanomastoidectomy; CWU: Canal wall up; CWD: Canal wall down.

Case no.	Reference	Year	Age	Sex	HPV	Malignancy	Recurrence	Final Therapy	FU
1	Stone et al. [3]	1987	55	M		Yes, SCC in the mastoid.	No	RM, RT	NED
2	Kaddour and Woodhead [4]	1992	77	F		No	No	D	No data
3	Seshul et al. [5]	1995	31	F		Yes, SCC in the nasal cavity, external auditory canal, mastoid.	No	RM, RT	AWD
4	Jones et al. [6]	1998	35	F	Positive (PCR)	Yes, carcinoma in the mastoid.	No	ERM	NED 14 months
5	Pou and Vrabec [7]	2002	81	M		Yes, SCC in the temporal bone. Carcinoma in situ in primary lesion.	Yes, after 6 months.	TBR	DOD 36 months
6			54	M		Yes, SCC in temporal bone and nasal cavity.	No	TBR, RT	NED 11 months
7	Kazuyuki et al. [8]	2009	65	M		No	Yes, after 2 months	RTM, RT	NED 10 months
8	Zhou et al. [9]	2011	52	M		Yes, carcinoma in situ in primary sinonasal and temporal lesion.	No	RM, TBR, RT	NED 8 months
9	Shen et al. [10]	2011	56	M		Yes, papillary SCC in the nasal cavity.	No	RM, RT	NED 6 months
10	Mitchell et al. [11]	2012	69	F		Yes, carcinoma in the sinonasal and temporal lesion	No	TBR, RT	NED 36 months
11	Dingle et al. [12]	2012	52	M		Yes, invasive SCC in the temporal lesion.	Yes, after 7 months.	RM, TBR, RT	AWD
12	Liu et al. [13]	2013	81	F	Positive	Yes, SCC in the sinonasal, middle ear, external auditory canal.	Yes, after 2 months and 4 months	Palliative RT	AWD
13	Haywood et al. [14]	2017	44	F	Positive	No	Yes, after 21 months	RM	NED 36 months
14	Current case	2016	76	M	Positive	Yes, SCC in the middle ear and mastoid.	Yes, after 20 months	CWU, CWD, RT	NED 5 months
